# Four recommendations to tackle the complex reality of transdisciplinary, natural experiment research

**DOI:** 10.3389/fpubh.2023.1240231

**Published:** 2023-10-18

**Authors:** Amber L. Pearson, Karin A. Pfeiffer, Rachel T. Buxton, Teresa H. Horton, Joseph Gardiner, Ventra Asana

**Affiliations:** ^1^CS Mott Department of Public Health, Michigan State University, Flint, MI, United States; ^2^Department of Kinesiology, Michigan State University, East Lansing, MI, United States; ^3^Department of Biology, Carleton University, Ottawa, ON, Canada; ^4^Department of Anthropology, Northwestern University, Evanston, IL, United States; ^5^Department of Epidemiology and Biostatistics, Michigan State University, East Lansing, MI, United States; ^6^National Coalition of Independent Scholars, Detroit, MI, United States

**Keywords:** nature-base solutions, greenspace, biodiversity, equitable, public health

## Abstract

Natural experiments are often used to study interventions in which randomization to control versus intervention conditions are impossible. Nature-based interventions (i.e., programs designed to increase human interaction with nature and improve human health) are commonly studied as natural experiments. We used a natural experiment design to explore the benefits of ecological rehabilitation of parks on biodiversity and resident health in low-income, minoritized neighborhoods in Detroit, MI. Given the complexities and interconnectedness of lived experiences, community needs, and ecological health, this research design has presented challenges. Based on our experiences, we pose four key recommendations for researchers and practitioners conducting natural experiments, nature-based interventions, and those working in low-income, minoritized neighborhoods. We use the explicit examples of challenges faced as rationale for these recommendations. The key recommendations are (1) Engage with community leaders; (2) Build a transdisciplinary team and work closely; (3) Examine privilege; and (4) Create a unified vision.

## 1. Introduction

Natural experiments are a robust alternative to Clinical Trials, particularly when randomizing to experience the intervention is not feasible or ethical (1). Many community-based interventions fall into this category. Community-based interventions that aim to empower communities and improve health equity are particularly complex. Rarely can intervention and controls be replicated, randomized, or stratified with ease and even when they can, the complexities of lived experiences can derail even the most well-planned experimental design. So, natural experiments offer a useful and cost-effective (2) alternative to understand the effect(s) of community-based interventions on residents. Moreover, exploring the outcomes of interventions along-side practitioners and grass-roots organizations can lead to increased uptake of findings into adaptive, evidence-based approaches (3).

Such knowledge sharing from real-world, community-based interventions studied using natural experiments is critical for improving the health of people and places with the highest need. Community-based interventions are on the rise, particularly nature-based ones. Nature-based interventions are programs that aim to increase human interaction with nature and/or restore an ecosystem to improve human health (4, 5). Epidemiological data show that people who live near natural areas, parks, and greenspaces have better health outcomes (6). But, natural experiments of nature-based interventions can be complex, given the need for research across disciplines to measure outcomes for biodiversity and human health.

Based on four years of natural experiment research, we outline four key recommendations for researchers and practitioners who plan to employ a natural experiment design to measure the health benefits of nature-based interventions, particularly in high need areas such as low-income, minoritized neighborhoods. We use experience from a study of an ecological park improvement intervention (7) in Detroit, MI, United States.

Detroit, similar to other post-industrial cities, has experienced formidable decline in its population. The city currently has a majority Black/African American population, comprising 78% of the city’s residents [using U.S. census terminology for race/ethnicity (8)] and high levels of poverty [32% (8)]. The population decline has led to countless abandoned buildings (9), vacant lots, and lower tax revenue mandating reductions in city services. Research has shown that, in the worst cases, vacancy can facilitate drug dealing and crime (10). Vacancy and lower tax revenue for city services, including the parks department, are challenges to Detroit but also serve as opportunities for potential low-cost land use alternatives such as ecological rehabilitation. However, the co-benefits of ecological rehabilitation (11) in urban areas for human and ecosystem health has gone largely unstudied (12), especially in the context of equity.

The intervention we studied involved ecological rehabilitation (i.e, removal of turf grass, litter, and broken cement, planting native species, installing trails, benches, and signage, and conducting engagement activities) of four parks (not being maintained as conventional parks) and surrounding vacant lots through a partnership between Detroit Parks and Recreation Department (DPRD) and Detroit Audubon (a bird conservation organization). The objective of the rehabilitation project was to transform unmaintained parks to habitat beneficial for birds. Our research team’s objective was to explore whether park rehabilitation, and resulting changes in biodiversity, influenced the health of neighboring residents. The original timeline involved rehabilitation in 2019–2020. However, the pandemic shifted the intervention to 2019 and 2021. The study is called the Study of Active Neighborhoods in Detroit, or StAND (7).

**Figure 1:**
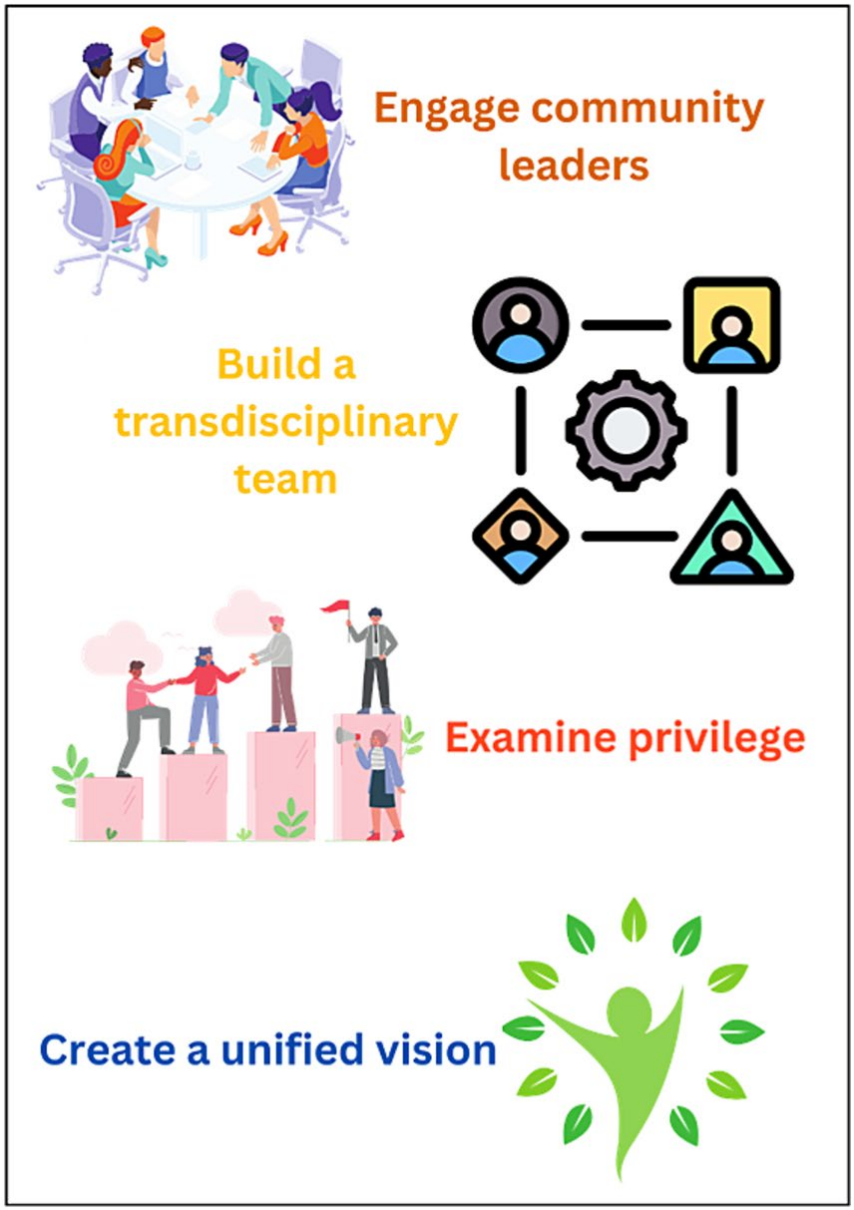
Four key recommendations for researchers and practitioners involved in nature-based solutions research.

Our transdisciplinary team of researchers aimed to measure changes in physical activity, stress, cardiometabolic health, and bird diversity over time, in both intervention and control neighborhoods [for more details, see Pearson et al. (7)] using a flexible longitudinal/repeated panel design. The team has expertise in kinesiology (Pfeiffer), biostatistics (Gardiner), ecotheology and community activism (Asana), geospatial techniques (Pearson), ecological acoustics and avian ecology (Buxton), and environmental physiology/endocrinology (Horton). Having completed four annual waves of data collection, here, we identify four key recommendations and provide rationale for these recommendations based on lessons learned over the course of conducting this research (Figure 1). These recommendations are for researchers conducting natural experiments or nature-based intervention research, and those working in places and populations with high need.

### 1.1. Engage community leadership

Community liaisons are frequently cited as highly important conduits in community-based interventions. Involving a liaison who is willing to engage in difficult conversations (with the residents, the practitioners, and the research team) can help the researchers have a pulse on the community, interpret research findings, and give the community a voice. The evidence is growing that community-engaged projects lead to better outcomes (13). However, consensus on what constitutes best practices for engagement has not been established (14). We recommend that prospective researchers invest time and effort in building meaningful and authentic relationships with the communities in which they plan to work, including attending community events, and staying informed of the activities of all partners (15, 16).

Organizations that seek strong nature-based interventions in heavily disinvested Black/African American communities must work with individuals from within the locus of that cultural community to identify culturally relevant interventions. Some data suggest that social equity issues make it challenging for resident involvement in greenspace improvements in marginalized communities (17). Working in low-income, predominantly minoritized areas often means that trust of White researchers is low [for good reasons, based on past harms (18)]. Our data collection required direct engagement with residents as a layer of engagement (1, 7). In preparation for this study (two years before funding was awarded), AP led a community workshop to make connections and listen to perspectives of residents on parks and greenspaces (19). From this experience, AP met VA, who has now worked as the Community Liaison for StAND since 2019. VA is a long-term resident of Detroit, with decades of experience in community organizing, and familiar with the communities to lead the engagement of the research team with between Detroit Audubon, DPRD, and residents.

We learned the value of following community leadership again and again. For example, the blinding of field staff and participants was an important part of the design in terms of neighborhood intervention versus control status for three reasons: (1) to minimize bias in the data, (2) to homogenize interactions with participants (whether conscious or unconscious), and (3) because ultimately carrying out the intervention was beyond the study team’s control so promising the intervention to any participant was deemed unethical. In our case, blinding was simply defined as non-disclosure of why each neighborhood was selected to be part of the study. During training each year, the investigators explained the concept of blinding to field staff. Training during the first year of the study provided a valuable learning experience. A few weeks into training, the investigators learned that the field staff did not fully understand blinding, which eroded their trust. Field staff questioned the motives of the investigators, as they perceived the investigators were holding back important information from participants. Under the leadership of the community liaison, the investigators worked to re-build rapport, establish credibility, and further explain blinding in research. Based on this experience, we modified future training sessions to better explain blinding up front and to anticipate and answer questions.

Another example involved ensuring that community leadership was central to the decisions of the practitioner, Detroit Audubon. The impact of the urban greening interventions often depends on acceptance or support by the residents (16, 20–22). Research on urban greening projects suggests that such interventions may be disruptive rather than beneficial when communities are not engaged in the design (16, 21). When working with conservation groups, the goals of rehabilitation may not align with community goals, and capacity for in-depth engagement is often low. Moreover, engagement in natural areas and birding in particular, are still predominantly White activities, where privilege and racism work to exclude non-White people (23). John James Audubon, the namesake of The Audubon Society and Detroit Audubon, was a prominent 19th century naturalist who used slaves to support his birding activities. Thus, care must be taken when restoring habitat for birds and bird watching in predominantly Black/African American neighborhoods. Although this layer of community engagement was the responsibility of Detroit Audubon, as a conservation organization they were not deeply experienced in community engagement. Thus, our Community Liaison advised Detroit Audubon in their engagement activities, including necessary incorporation of culture-specific considerations pertaining to Black/African American perceptions of greenspace improvements. Particularly, these perceptions about abandoned park spaces relate to whether park improvements would even take place, and whether there is even a need for nature rehabilitation for birds. Rooted in an historical distrust of promised improvements based upon memories of deliberate urban disinvestment in Black/African American neighborhoods in Detroit, Michigan, it proved challenging to identify and recruit community stakeholders for buy-in to support ecological rehabilitation in one heavily disinvested community. Despite numerous in-person and virtual presentations with detailed information about proposed improvements, interest flagged over many months. Further, the COVID-19 pandemic exacerbated the problem of strong community buy-in, resulting in almost no interest in the proposed upcoming park improvements.

### 1.2. Build a transdisciplinary team of experts and work closely

A transdisciplinary approach that integrates eco-theology, community activism, and community-based participatory research with ecology and public health may be important in particular communities and can provide avenues to better facilitate stakeholders’ participation. Early in the design process, engage with experts across disciplines to ensure that the methodology to measure both ecological and health changes is robust. Often community engagement specialists, ecologists and public health or social scientists do not “speak the same language” and time and effort is required to make sure all disciplines can work toward complementary goals.

Evaluating the co-benefits of urban ecological rehabilitation projects that aim to benefit both humans and biodiversity of plants, birds, and insects requires working across disciplines and institutions. Any trade-offs between conservation and community goals should ultimately be resolved through community guidance. We have found that despite mounting interest in interdisciplinary work to tackle “wicked problems” of the 21st century, few projects exist to act as a model for our own research. For example, in an assessment of the effectiveness of nature-based solutions in urban areas, one review found only two studies that explored co-benefits, or both environmental and social or health-related outcomes (24, 25). Thus, before embarking on our research program, we were unable to draw from previous experiences to explore successes and challenges of interdisciplinary research on the outcomes of natural experiments or coupled human and natural systems.

Our intention was to measure health outcomes pre- and post-intervention, however, if the benefits of restoration are delivered through increased species diversity and slowly developing vegetation cover, it is difficult to determine which time periods represent these analytical cut-points. Most urban environments are “novel ecosystems” – fundamentally different from natural ecosystems (26). Thus, understanding goals and benchmarks for restoration is challenging and restoration outcomes are difficult to predict (27, 28). In our case, soil quality and seed banks varied widely among the restoration sites, where weeds grew much better at sites with high quality soil, making establishment of native species challenging. These sites will require more consistent mowing and tilling, and limited resources are available for intensive upkeep. Moreover, these complexities prohibit clear delineation of an “intervention year.” While urban ecosystems may not be identical to more pristine ones, there are measureable changes and benefits of their rehabilitation. Clearly measuring the benefits of rehabilitation for biodiversity and humans will help illuminate the pathways through which nature-based interventions best improve health, and for whom.

Beyond diversity among the investigative team, we recommend engagement with the conservation experts and practitioners leading the nature-based intervention. By their very definition, natural experiments are not within the control of the researcher. This can pose threats to study design and timing as priorities or funding may change for the interventionists, which can lead to fewer sites receiving the intervention, a revised form of the intervention, or delays. These changes may even occur after baseline data are collected, incurring costs to the research study and misbalancing the intervention versus control recruitment targets. For example, our protocol reported the intention to collect data for five intervention and five control sites, while we currently have four intervention sites and seven control sites for reasons beyond the research team’s control (7). Audubon Detroit and DRPD shifted their priorities away from restoring small, neighborhood parks and changed the location of an intervention park. This decision led to an imbalance in the number of control and intervention neighborhoods in our study. Another example was the postponement of the intervention due to the COVID-19 pandemic (particularly prior to vaccine availability). The postponement resulted in less follow-up time post-intervention. Because the intervention under study is an ecological rehabilitation, this has implications for plant maturation, biodiversity changes for avian, insect, and plant species, and ultimately the ways in which people use and benefit from restored spaces.

Changes in implementation of the intervention require research teams to be flexible in designing statistical analyses and can result in analyses not strictly following previously published protocols (7). Staying in communication and working closely with the intervention experts is essential, in order to pivot and make decisions that affect study design, staffing, and recruitment targets in real time. In practice, this means holding regular meetings and keeping accurate minutes from each meeting.

### 1.3. Examine privilege

It can be difficult for research teams to truly understand what is happening in communities of interest due to unintentional, implicit bias. Researchers and practitioners need to be open to realizing when they make missteps in the eyes of community members and mending situations with as much grace as possible. At times, the field staff on the research team may also exhibit privilege in the eyes of participants and residents in the nature-based intervention neighborhoods.

For example, our staff and participants faced safety and trust issues in both directions. At times, staff felt unsafe in certain parts of neighborhoods or working with particular participants. In fact, one neighborhood was ultimately deemed too unsafe for staff, and we did not conduct further recruitment after the first few weeks. We intentionally attempted to hire only staff who were Detroit residents, with the aim of ensuring that staff had contextual experience in Detroit neighborhoods and the realities of our participants’ lives. While this was possible for most staff, we also had to hire some individuals who were from suburbs of the city. In many cases, we lost those staff members relatively quickly, as most did not feel comfortable in the environments where data were being collected. In the other direction, at times participants did not trust staff members upon their initial interaction. For most participants, this waned quickly through conversation. A Community Liaison can prove very useful in highlighting when optics are suboptimal or when privilege may be limiting the ability to successfully carry out the research and/or the intervention.

### 1.4. Create a unified vision

Exploring the outcomes of natural experiments is complex from an ecological conservation (29) and public health (30) perspective; thus, exploring both simultaneously results in amplified challenges. In urban environments, restoring ecosystem functioning is complex. For community buy in, ensuring that the research aims and the intervention prioritizes human benefits and considerations is essential. While many conservation groups are rightly focused on the benefits to biodiversity, the intervention is unlikely to succeed or endure time if community needs are not central to the intervention design and messaging.

## 2. Conclusion

Natural experiments offer a rigorous design, and perhaps the only realistic way to measure the co-benefits of nature-based interventions in communities. Because natural experiments happen under real-world conditions, they are inherently complex and involve multiple layers of perspectives, values, and trade-offs. So, natural experiments examining the nature-based interventions face challenges, which we report as four key recommendations for the research design, practice, and dissemination stages to bolster success. The key recommendations are (1) Engage community leadership, (2) Engage a transdisciplinary team and work closely; (3) Examine privilege; and (4) Create a unified vision. Despite these challenges, the rewards of conducting this complicated research may be far-reaching, including the genuine synergy of conservation biology, public health, and social science research to benefit both human and planetary health – a true “nature-based solution”.

## Data availability statement

The original contributions presented in the study are included in the article/supplementary materials, further inquiries can be directed to the corresponding author.

## Author contributions

AP, KP, RB, TH, JG, and VA conceived of the manuscript. AP, KP, RB, and TH drafted the manuscript. All authors contributed to the article and approved the submitted version.

## Funding

This study was funded by the National Cancer Institute (NCI) of the National Institutes of Health (NIH) under grant R01CA239197 (Pearson), the Detroit Medical Center, Michigan State University’s Clinical Translational Science Initiative, the Vice President for Research and Graduate Studies and the Provost Undergraduate Research Initiative. Salary for TH is provided by the Negaunee Foundation.

## References

[1] Leatherdale ST. Natural experiment methodology for research: a review of how different methods can support real-world research. Int J Soc Res Methodol. (2019) 22:19–35. doi: 10.1080/13645579.2018.1488449

[2] Branas CC Kondo MC Murphy SM South EC Polsky D MacDonald JM. Urban blight remediation as a cost-beneficial solution to firearm violence. Am J Public Health. (2016) 106:2158–64. doi: 10.2105/AJPH.2016.303434, PMID: 27736217 PMC5104992

[3] Crane M Bohn-Goldbaum E Grunseit A Bauman A. Using natural experiments to improve public health evidence: a review of context and utility for obesity prevention. Health Res. Policy Syst. (2020) 18:48. doi: 10.1186/s12961-020-00564-2, PMID: 32423438 PMC7236508

[4] Tambyah R Olcoń K Allan J Destry P Astell-Burt T. Mental health clinicians’ perceptions of nature-based interventions within community mental health services: evidence from Australia. BMC Health Serv Res. (2022) 22:841. doi: 10.1186/s12913-022-08223-8, PMID: 35773704 PMC9244442

[5] Cohen-Shacham E.., Nature-based solutions to address global societal challenges. (2016), Gland, Switzerland: International Union for the Conservation of Nature. xiii–97.

[6] Twohig-Bennett C Jones A. The health benefits of the great outdoors: a systematic review and meta-analysis of greenspace exposure and health outcomes. Environ Res. (2018) 166:628–37. doi: 10.1016/j.envres.2018.06.030, PMID: 29982151 PMC6562165

[7] Pearson AL Pfeiffer KA Gardiner J Horton T Buxton RT Hunter RF. Study of active neighborhoods in Detroit (StAND): study protocol for a natural experiment evaluating the health benefits of ecological restoration of parks. BMC Public Health. (2020) 20:638. doi: 10.1186/s12889-020-08716-3, PMID: 32380967 PMC7204306

[8] US Census. QuickFacts: Detroit city, Michigan. 2020-2021; Available at: https://www.census.gov/quickfacts/fact/table/detroitcitymichigan/BZA115221.

[9] Dewar M Seymour E Druță O. Disinvesting in the City: the role of tax foreclosure in Detroit. Urban Aff Rev. (2014) 51:587–615. doi: 10.1177/1078087414551717

[10] Garvin EC Cannuscio CC Branas CC. Greening vacant lots to reduce violent crime: a randomised controlled trial. Inj Prev. (2013) 19:198–203. doi: 10.1136/injuryprev-2012-040439, PMID: 22871378 PMC3988203

[11] Liu J Dietz T Carpenter SR Taylor WW Alberti M Deadman P. Coupled human and natural systems: the evolution and applications of an integrated framework. Ambio. (2021) 50:1778–83. doi: 10.1007/s13280-020-01488-5, PMID: 33721224 PMC7957461

[12] Colléony A Shwartz A. Beyond assuming co-benefits in nature-based solutions: a human-centered approach to optimize social and ecological outcomes for advancing sustainable urban planning. Sustainability. (2019) 11:4924. doi: 10.3390/su11184924

[13] Hudson D Gilbert K Goodman M. Promoting authentic academic&mdash;community engagement to advance health equity. Int J Environ Res Public Health. (2023) 20:2874. doi: 10.3390/ijerph20042874, PMID: 36833570 PMC9957457

[14] O’Mara-Eves A Brunton G Oliver S Kavanagh J Jamal F Thomas J. The effectiveness of community engagement in public health interventions for disadvantaged groups: a meta-analysis. BMC Public Health. (2015) 15:129. doi: 10.1186/s12889-015-1352-y, PMID: 25885588 PMC4374501

[15] Why am I always being researched. (2019); Available at: https://chicagobeyond.org/researchequity/.

[16] Campbell-Arvai V Lindquist M. From the ground up: using structured community engagement to identify objectives for urban green infrastructure planning. Urban For Urban Green. (2021) 59:127013. doi: 10.1016/j.ufug.2021.127013

[17] Shanahan DF Astell–Burt T Barber E Brymer E Cox D Dean J. Nature–based interventions for improving health and wellbeing: the purpose, the people and the outcomes. Sports. (2019) 7:141. doi: 10.3390/sports7060141, PMID: 31185675 PMC6628071

[18] Skloot R. The immortal life of Henrietta lacks, vol. 2010. New York: Random House Audio (2010), Unabridged). 2010 p.

[19] Matthews AD. Community members' perspectives on a community-engaged process for supporting vibrant greenspaces in Detroit. J Commun Engag High Educ. (2022) 14:26–41.

[20] Kim G Newman G Jiang B. Urban regeneration: community engagement process for vacant land in declining cities. Cities. (2020) 102:102730. doi: 10.1016/j.cities.2020.102730, PMID: 32831449 PMC7440045

[21] Triguero-Mas M Anguelovski I García-Lamarca M Argüelles L Perez-del-Pulgar C Shokry G. Natural outdoor environments’ health effects in gentrifying neighborhoods: disruptive green landscapes for underprivileged neighborhood residents. Soc Sci Med. (2021) 279:113964. doi: 10.1016/j.socscimed.2021.113964, PMID: 34020160

[22] Anguelovski I Connolly JJT Cole H Garcia-Lamarca M Triguero-Mas M Baró F. Green gentrification in European and north American cities. Nat Commun. (2022) 13:3816. doi: 10.1038/s41467-022-31572-1, PMID: 35780176 PMC9250502

[23] Cashman-Brown O. Birds of a feather: the whiteness of birding In: Falkof N Cashman-Brown O, editors. On whiteness. Leiden, The Netherlands: Brill (2012). 173–82.

[24] Dumitru A Frantzeskaki N Collier M. Identifying principles for the design of robust impact evaluation frameworks for nature-based solutions in cities. Environ Sci Pol. (2020) 112:107–16. doi: 10.1016/j.envsci.2020.05.024

[25] Luo Y Lü Y Fu B Zhang Q Li T Hu W. Half century change of interactions among ecosystem services driven by ecological restoration: quantification and policy implications at a watershed scale in the Chinese loess plateau. Sci Total Environ. (2019) 651:2546–57. doi: 10.1016/j.scitotenv.2018.10.116, PMID: 30340190

[26] Teixeira CP Fernandes CO. Novel ecosystems: a review of the concept in non-urban and urban contexts. Landsc Ecol. (2020) 35:23–39. doi: 10.1007/s10980-019-00934-4

[27] Klaus VH Kiehl K. A conceptual framework for urban ecological restoration and rehabilitation. Basic Appl Ecol. (2021) 52:82–94. doi: 10.1016/j.baae.2021.02.010

[28] Klaus VH. Urban grassland restoration: a neglected opportunity for biodiversity conservation. Restor Ecol. (2013) 21:665–9. doi: 10.1111/rec.12051

[29] Ockendon N Amano T Cadotte M Downey H Hancock MH Thornton A. Effectively integrating experiments into conservation practice. Ecol Solution Evid. (2021) 2:e12069. doi: 10.1002/2688-8319.12069

[30] Craig P Katikireddi SV Leyland A Popham F. Natural experiments: an overview of methods, approaches, and contributions to public health intervention research. Annu Rev Public Health. (2017) 38:39–56. doi: 10.1146/annurev-publhealth-031816-044327, PMID: 28125392 PMC6485604

